# Reversing Type 2 Diabetes: The Time for Lifestyle Medicine Has Come!

**DOI:** 10.3390/nu12071974

**Published:** 2020-07-03

**Authors:** Isabelle Lemieux

**Affiliations:** Centre de Recherche de l’Institut Universitaire de Cardiologie et de Pneumologie de Québec—Université Laval, Québec, QC G1V 4G5, Canada; isabelle.lemieux@criucpq.ulaval.ca; Tel.: +1-418-656-8711 (ext. 3603)

The IDF (International Diabetes Federation) Diabetes Atlas Committee has recently published the global estimates of diabetes prevalence for 2019 [1]. Sadly but not surprisingly, these latest statistics indicate that the prevalence of diabetes has reached 9.3% (463 million people). Moreover, the forecasts are hardly optimistic as it was estimated that the prevalence of diabetes will increase to 10.2% (578 million) by 2030 and 10.9% (700 million) by 2045 [1]. Considering that type 2 diabetes accounts for nearly 90% of total diabetes cases, needless to say we must take this matter seriously. 

Type 2 diabetes is a societal chronic disease associated with several macrovascular and microvascular problems, with cardiovascular complications being the leading cause of morbidity and mortality in these patients [2]. Thus, preventing and optimally managing type 2 diabetes is critical to reduce the health burden associated with this disease. In this regard, the close relationship between overweight/obesity and the risk of developing type 2 diabetes is now well established [3]. However, with the development of imaging techniques such as computed tomography and magnetic resonance imaging, it has become evident that the link between obesity and type 2 diabetes is not explained by excess body fatness per se. For instance, numerous cardiometabolic imaging studies published in recent years have revealed that excess visceral adipose tissue is an independent risk factor for the development of type 2 diabetes even after controlling for total body fatness [4]. More recently, other ectopic fat depots such as liver fat and pancreatic fat have also been shown to be closely related to type 2 diabetes [5]. 

Although treating type 2 diabetes complications is of utmost importance to protect organ damage, it is now increasingly recognized that even the more ambitious goal of diabetes remission may be possible [6]. Thus, while there is little doubt that healthy lifestyle habits are the cornerstone of prevention of type 2 diabetes, they could also be used as an effective treatment to even potentially reverse type 2 diabetes. In this regard, the interesting narrative review published by Hallberg SJ and colleagues [7] has summarized the evidence that type 2 diabetes reversal is possible with the use of three different approaches: bariatric surgery, low-calorie diets and carbohydrate restriction. Bariatric surgery has systematically produced a significant reduction in blood glucose [8] as well as a decrease in hypoglycemic medications and a remission of type 2 diabetes in up to 80% of patients [9,10,11]. Although very effective in the short term, long-term reversal of type 2 diabetes with bariatric surgery remains unclear. Moreover, this type of intervention is obviously associated with surgical complications. 

This review [7] also elegantly presented evidence of the effectiveness of low-calorie diets to generate type 2 diabetes reversal. While initial studies mostly documented improvements in glycemic control with low-calorie diets rather than diabetes remission, more recent studies have examined the long-term effect of low-calorie diets on type 2 diabetes remission [12,13,14]. In the Look AHEAD trial, for instance, the remission of type 2 diabetes was greater in type 2 diabetic patients in the intensive lifestyle intervention arm (reduction in calorie intake and increase in physical activity level) than in the control group [15]. As for bariatric surgery, the long-term reversal of type 2 diabetes appears to remain a challenge. 

Finally, the last approach discussed in the narrative review of Hallberg [7] was low-carbohydrate diets which were the most frequently prescribed treatment for diabetes before the discovery of insulin [16]. Although there was little interest in that type of diet until recently, new evidence on the efficacy of low-carbohydrate diets in the management of type 2 diabetes has convinced scientific societies to include this diet in their treatment algorithms [17]. However, long-term data are lacking to prove its efficacy. Thus, Hallberg’s excellent narrative review [7] gathers important information on the relevance of bariatric surgery and two different types of diet as tools to induce type 2 diabetes remission. 

However, one aspect of diabetes management, remission and prevention has not received enough attention in this nice article: physical activity/exercise. Physical activity and exercise are not only effective to prevent the development of type 2 diabetes in high-risk patients, but these behaviours are also useful to treat patients with type 2 diabetes and even lead to remission in some cases. Through several molecular mechanisms, regular physical activity/exercise can delay or prevent the development of type 2 diabetes [18,19,20,21]. Combined with a reduction in calorie intake and weight loss, increasing physical activity has been associated with type 2 diabetes remission [12,15,22]. An improvement in blood glucose levels in type 2 diabetes, even in the absence of weight loss, has also been reported [23]. Subgroup analyses of the Diabetes Remission Clinical Trial have elegantly shown that ectopic fat mobilization, especially liver fat and pancreatic fat, are closely related to the development of diabetes as well as its reversal [24,25]. Not only can physical activity/exercise acutely deplete glycogen stores, it can eventually chronically improve insulin sensitivity, thereby favourably preserving beta-cell function [26,27]. In addition, regular physical activity/exercise has been shown to induce a selective mobilization of visceral adipose tissue and liver fat, two key drivers of the insulin-resistant-hyperglycemic state of type 2 diabetes, these favourable effects being observed even in the absence of weight loss [28]. Thus, considering that the beneficial effects of physical activity/exercise in patients with type 2 diabetes go way beyond improvements in glycemic control, our healthcare systems should optimize type 2 diabetes management and consider the implementation of structures such as those implemented in cardiac rehabilitation programs [29]. 

In conclusion, a diversity of personalized solutions, which must include a high-quality nutritional diet and a physically active lifestyle, along with other important lifestyle determinants such as alcohol consumption, smoking and sleeping habits, should be promoted in order to curb the type 2 diabetes epidemic sweeping the world. Together with population-based solutions that will lead to the development of environments promoting healthy behaviours, lifestyle medicine [30] should help to meet the Sustainable Development Goal target 3.4 adopted by the UN Member States to reduce premature mortality from chronic diseases through prevention and treatment by 2030 [31].
